# Health and social challenges faced by rare disease patients in Hong Kong: a cross-sectional study

**DOI:** 10.1186/s13023-026-04482-4

**Published:** 2026-07-24

**Authors:** Roger Yat-Nork Chung, Alvin Yik-Kiu Hui, Dong Dong, Jonathan Ka-Long Mak, Cheryl Shu Ming Chia

**Affiliations:** 1 JC School of Public Health and Primary Care, The Chinese University of Hong Kong, Hong Kong, SAR China; 2 CUHK Centre for Bioethics, The Chinese University of Hong Kong, Hong Kong, SAR China; 3 CUHK Institute of Health Equity, The Chinese University of Hong Kong, Hong Kong, SAR China; 4 Institute of Public Health and Wellbeing, University of Essex, England, UK; 5 Department of Medical Epidemiology and Biostatistics, Karolinska Institutet, Stockholm, Sweden; 6 Department of Pharmacology and Pharmacy, The University of Hong Kong, Hong Kong, SAR China; 7 Department of Orthopaedics and Traumatology, The Chinese University of Hong Kong, Hong Kong, SAR China

**Keywords:** Rare disease, Healthcare needs, Treatment access, Health-related quality of life, Hong Kong

## Abstract

**Background:**

Literature suggests that rare disease (RD) have adverse effects on patients’ physical and psychosocial well-being. RD patients also face various challenges in their daily lives. This study aims to quantify the impact of RDs on patients in Hong Kong in terms of their sociodemographic characteristics, health-related conditions, patient experience, psychosocial support, illness perception, and self-esteem.

**Methods:**

We conducted a cross-sectional survey of RD patients in Hong Kong. A mixed-mode survey approach, which included purposive, opportunity, and snowball sampling, was adopted. Participants were recruited through Rare Disease Hong Kong (RDHK) and completed a face-to-face, interviewer-administered survey(*n* = 176). The 127-item survey instrument covered aspects on sociodemographic characteristics, health-related conditions, patient experience, psychosocial support, illness perception, and self-esteem. Health-related quality of life (HRQL) was assessed using 12-item Short-Form Health Survey version 2 (SF-12 v2), from which a Physical Component Summary was derived for physical health, and a Mental Component Summary for psychological health. Univariate analyses and multivariate linear regression analyses were conducted subsequently to examine the associations between HRQL and sample characteristics.

**Results:**

RD patients in this study were found to have poor physical and psychological HRQL compared with population norms. They also faced challenges and difficulties in their daily lives, including but not limited to economic activities, ability to work, sleep quality, activities of daily living, patient experience, psychosocial support, outlook on life, and self-esteem.

**Conclusions:**

To our knowledge, this is the first study in Hong Kong that quantifies the impact of RDs on patients’ HRQL, and their experience throughout the disease journey. Due to the multi-faceted impact of RD on their patients, we opine that it is critical to adopt a capability approach that does not merely emphasize on the resourcist right and entitlement of the patients, but also ensures equality in capabilities in converting resources to outcomes they value by focusing on the social determinants of health beyond healthcare policies.

**Supplementary Information:**

The online version contains supplementary material available at https://doi.org/10.1186/s13023-026-04482-4.

## Introduction

### Background

Rare disease (RD) is a large and diverse group of diseases with a low prevalence in the population, some of which are life-threatening and chronically debilitating [1]. There is an estimate of 6,000 to 8,000 RDs [2] which could collectively affect 3.5% to 5.9% of all people in the world [3]. Recent estimates suggest that over 300 million people globally are living with rare disease, representing a substantial global public health burden [3]. RDs have various definitions across different countries [4, 5]. For instance, RDs in the US are defined by the number of affected people, while RDs in Europe and Taiwan are defined by its prevalence rate [6, 7]. No official definition or operational boundary of RDs had been established in Hong Kong Special Administrative Region (SAR) of China. To date, one study using hospital admission data estimated that one in 67 (1.5%) of the population in Hong Kong was affected by RDs, which was approximately 109,535 people [8]. Owing to the rarity of the diseases and the usually lack of treatments, RDs could pose significant impact on the physical and psychological health-related quality of life (HRQL) of their patients [9].

Various studies have explored the impact of RDs on patients’ HRQL. It is commonly reported that RD patients suffer from poor physical and mental health [10, 11], and the health impact is dependent on diseased-related factors such as degree of frequency of medical complications and disability and impairment [9]. Consistent with previous research, RD patients tend to have poorer HRQL when compared to the general population [11–14], and those who suffered from more than one chronic disease at the same time tended to have lower HRQL [11, 14]. However, research has also shown that the severity or the physical manifestations of RDs is not the only contributor to a poorer HRQL; instead, RD patients’ HRQL could also be affected by other non-clinical factors such as psychosocial support, illness perceptions, and coping strategies [9]. Specifically, studies have found that RD patients with more fatalistic or helpless attitudes tend to have lower HRQL [9], while those with greater acceptance, optimism and hopefulness tend to have higher HRQL [15]. On the other hand, evidence indicates that RD patients who are depressed, anxious and with persistent chronic symptoms tend to have lower HRQL, and these factors can negatively affect the course of the chronic illness [16].

Apart from the physical and psychological well-being of RD patients, RD may also affect their healthcare experiences over their disease journey. For example, one previous survey conducted in Australia revealed that 30% of the respondents had to wait for five or more years for a diagnosis, and 46% of them had received at least one misdiagnosis [17]. Another study suggested that on average it took 7.6 years in the US and 5.6 years in the United Kingdom (UK) for RD patients to receive a proper diagnosis, and the RD patients were subjected to two to three misdiagnoses [10]. A recent global patient and caregiver survey similarly found that diagnostic delays remain a persistent challenge, with the average time from the first onset of symptoms to a confirmed diagnosis taking up to 4.7 years on average, and 25% of respondents reporting having received eight or more consultations with a healthcare professional before containing a diagnosis confirmation [18]. Moreover, several studies also showed that RD patients often receive no or limited information on their RD(s) [10, 11, 19]. Research has also demonstrated that they also tended to receive insufficient social and psychological support [17, 19], which means that they may not be able to gather information and resources from peer support groups [19], thereby negatively affecting their HRQL.

The Hong Kong government provides support to RD patients mainly through two means, namely diagnostic services and financial support. In terms of diagnostic services, the Clinical Genetic Service of the Department of Health (DH) provides clinical diagnosis, counselling, and prevention services for families that are potentially affected by genetic-related diseases, while the Hospital Authority (HA), the statutory body responsible for managing all public hospitals services and clinics in Hong Kong, provides medical services for patients suffering from uncommon disorders [20]. Moreover, HA also provides two funds, namely the Samaritan Fund (SF) and the Community Care Fund (CCF) Medical Assistance Programmes, to financially support patients who need self-financed items (SFI) that are not covered by the HA’s standardised items required in the course of their medical treatments. The SF covers expenses for Privately Purchased Medical Items (PPMI) and self-financed drugs with proven effectiveness [21], while the CCF covers ultra-expensive drugs for treating uncommon disorders with less but rapidly increasing clinical evidence of effectiveness [22]. As of March 2021, the number of drugs covered by the SF and CCF Medical Assistance Programmes was 51 and 37 respectively [23].

In the context of Hong Kong, while there are several government documents outlining the difficulties faced by RD patients throughout their disease journey [24–26], and a quantitative study examining the EQ-5D-3-level of RD patients, which showed significant association between lower HRQL with neurologic diseases, high out-of-pocket expenditure, home modification, and living in public housing or subdivided flats/units [27], no quantitative study has comprehensively investigated the physical, mental and psychosocial domains of HRQL of RD patients, as well as the actual challenges and difficulties experienced among them in this Chinese city. These difficulties typically include uncertainty with regards to delayed diagnosis, unavailability of treatments, and/or limited access to costly drugs and treatments, to name a few. Therefore, it is crucial to study both the physical and psychological aspects of RD patients’ HRQL, as well as their experience in dealing with their RDs. Moreover, most studies on RDs are clinical trials with small sample size [28], and focused on one single type of RD rather than looking into different types of RDs as an entity. By identifying patients with different rare conditions as an entity due to their shared patient experiences and filling in this research gap, findings of our present study can contribute to the literature and inform measures to improve RD patients’ HRQL.

## Aims and objectives

This study aims to quantify the impact of RDs on patients using patient reported outcome measures, and to identify their needs in terms of their daily living activities, patient experience, physical, emotional and psychosocial health, and patients’ healthcare access and utilisation pattern. Specifically, the objectives of this study are: (1) to quantify the sociodemographic characteristics, health-related conditions, patient experience, psychosocial support, illness perception, and self-esteem of RD patients; and (2) to examine the association between RD patient characteristics and physical and psychological HRQL.

## Methods

### Study design and participants

Data were collected from a cross-sectional survey of RD patients in Hong Kong. Given the low prevalence of each individual RD and the absence of a comprehensive sampling frame of RD patients in the city, a mixed-mode survey approach, including purposive, opportunity, and snowball sampling, was adopted to recruit as many participants as possible. Between 9 April and 2 September 2018, a total of 176 individuals were successfully recruited to complete a face-to-face, interviewer-administered structured survey. The recruitment was facilitated through the network and promotion of Rare Disease Hong Kong (RDHK; formerly Hong Kong Alliance for Rare Diseases), a charitable institution recognized by the Hong Kong government. RDHK is the first patient group in Hong Kong comprising patients and their families across different rare diseases. The survey interviews were conducted face-to-face by trained research assistants under the supervision of the Principal Investigator. All research assistants held at least a bachelor’s degree and completed a training program led by the Principal Investigator, which covered the survey instrument, standardized interview protocols, ethical considerations, and data collection procedures. Training included role-playing exercises and supervised practice interviews to ensure consistency in survey administration. Interviews were conducted at locations agreed upon by the participants, primarily in private rooms within the offices of the patient organizations with which the participants were affiliated. Registrations for the survey were accepted via telephone, email, instant messengers (e.g. WhatsApp, Facebook), and through referrals from other rare disease patient organizations, public clinics, and hospitals. A total of a total of 24 organizations and units referred participants for the survey, as listed in Appendix Table 1. The survey took approximately 30 to 45 min to complete. Inclusion criteria were RD patients who were Hong Kong residents aged 18 or above who spoke Cantonese. One individual did not complete the entire interview, resulting in a final sample size of *n* = 176. With a previous estimate of about 110,000 rare disease patients in Hong Kong [8], our study has sampled approximately 0.16% of this population. Nevertheless, with the absence of an official comprehensive patient registry in Hong Kong, our study is important in shedding light on the health and social challenges faced by rare disease patients in Hong Kong even with a relatively small sample size.

**Table 1:** Rare disease groups based on linearized Orphanet classification (*n* = 176)

Disease groups	Number of individuals	%
Rare neurologic disease	91	48.1
Rare developmental anomaly during embryogenesis	34	18.0
Rare eye disease	21	11.1
Rare systemic or rheumatologic disease	12	6.3
Rare bone disease	11	5.8
Rare inborn errors of metabolism	8	4.2
Rare hematologic disease	6	3.2
Rare skin disease	2	1.1
Rare neoplastic disease	1	0.5
Rare respiratory disease	1	0.5
Rare immune disease	1	0.5
Unclassified	1	0.5
Total	189^	100

^ This exceeds 176 since 163 (92.6%) had reported having one RD, while 13 (7.4%) had two RDs at the same time

Prior to the start of the interviews, all participants were explained the details of the study, their rights as participants, and asked to sign an informed consent form. Ethical approval had been obtained from the Survey and Behavioral Research Ethics Committee of the Chinese University of Hong Kong.

### Measurements

The survey instrument contained 127 questions, covering aspects on sociodemographic and socioeconomic characteristics, health-related condition, patient experience, psychosocial support, illness perception, and self-esteem. The questions were formulated by the research team based on a related survey [29] with incorporation of several validated scales as follows.

#### Sociodemographic and socioecoomnic characteristics

We collected information on their sex, age, marital status, education, employment status and occupation, monthly household income, type of housing, and whether they had domestic helper. We also directly asked whether they did not receive adequate treatment due to financial problem.

#### Health-related conditions

Health-related quality of life (HRQL) was examined using a validated Chinese version of the 12-item Short-Form Health Survey version 2 (SF-12 v2) [30]. It consists of 12 questions, measuring eight health domains of physical (general health, physical functioning, role-physical, body pain) and psychological health (vitality, social functioning, role-emotional, psychological health). After calculating scores in each domain, two distinct summary scores were derived, including Physical Component Summary (PCS) for physical health and Mental Component Summary (MCS) for psychological health. A norm-based scoring algorithm with reference to data from a Hong Kong general population survey was applied [31, 32]. Both scores range from 0 to 100, where higher scores indicate better physical and psychological health of the individual. PCS and MCS were used the outcome measures for examining the associations of RD patient characteristics with their physical and psychological HRQL.

A Chinese version 10-item Barthel Index was used to assess activities of daily living (ADL) [33]. It includes a total of 10 domains (stairs, walking, transfers, toilet use, bladder control, bowel control, dressing, grooming, bathing, and feeding), from which participants were categorized into five levels of independence according to the total ADL score. They include total dependence (score 0–20), severe dependence (score 21–60), moderate dependence (score 61–90), slight dependence (score 91–99) and independence (score 100).

A Cantonese version 19-item Pittsburgh Sleep Quality Index (PSQI) was adopted to assess sleep quality [34]. It includes seven components, namely subjective sleep quality, sleep latency, sleep duration, habitual sleep efficiency, sleep disturbances, use of sleep medication, and daytime dysfunction. A total PSQI score, ranging from 0 to 21, was obtained by summing up all the component scores, in which a higher score indicates poorer sleep quality.

Disability in community participation was assessed by the Chinese version 8-item of the “participation in society” domain of the World Health Organization Disability Assessment Schedule (WHODAS) 2.0 [35], with each question rated on a five-point scale. A total domain score ranging from 0 to 100 was calculated, where a higher score indicates worse functioning.

In addition, we collected information on their chronic conditions, from which we derived the number of chronic diseases (including their rare diseases) for analysis.

#### Patient experience

We collected information on their age of diagnosis, time taken and number of specialists consulted to confirm diagnosis, number of misdiagnosis, experiences of uncertainties and despair about the diagnosis, whether they received enough information on disease management, difficulty in receiving treatment for the RD and for managing symptoms, as well as satisfaction with the treatment received.

#### Psychosocial support

A Chinese version 12-item Multidimensional Scale of Perceived Social Support (MSPSS) was applied to estimate social support level received from family, friends, and significant other [36]. A higher total score indicates a higher level of social support.

Also, we asked whether they felt being treated friendly, understood, and respected by other people, and whether their relationships with family and friends had suffered due to their rare disease.

#### Illness perception

We directly asked whether they were worried about their future health, whether they felt hopeful that their diseases would be cured in the future, and whether they accepted their illness and had a positive outlook on life.

#### Self-esteem

A Chinese version 10-item Rosenberg Self-esteem Scale (RSE) was incorporated to examine self-esteem of individuals [37, 38]. Scores in each item were recoded and added up to yield a total score. Individuals were categorized into three groups: low self-esteem (score < 15); within normal range (score 15–25); and high self-esteem (score > 25).

### Statistical analysis

Types of RD of participants were categorized based on Orphanet’s linearization rules [39]. Sample characteristics were summarized as proportions for categorical variables and mean ± standard deviation (SD) for continuous variables. Univariate analyses of continuous Physical Component Summary (PCS) and Mental Component Summary (MCS) scores with participants’ characteristics were performed by t-tests or one-way ANOVA for categorical variables, and by Pearson or Spearman correlation tests for continuous variables, depending on their distribution and normality. Variables with *p* < 0.1 were included in the subsequent multivariable linear regression models to assess their independent associations with PCS and MCS scores. β-coefficients with 95% confidence intervals (CI) were reported, in which a positive coefficient represents better physical or psychological health status, and vice versa. R-squared was calculated for linear regression models to assess the proportion of variation explained by the independent variables. All the statistical analyses were performed using Stata 14.2. We followed the STROBE cross-sectional studies checklist for our study reporting [40].

## Results

### Type of RD

Of the 176 respondents, 163 (92.6%) had reported having one RD, while 13 (7.4%) had two RDs at the same time. The most common type of RD found in the sample was rare neurologic disease (48.1%), followed by rare developmental anomaly during embryogenesis (18.0%), and rare eye disease (11.1%). Please refer to Table 1 for broad categories of the RDs, and Appendix Table 2 for all the RDs included in the analysis of this study.

**Table 2:** Physical and mental component summary scores by characteristics of participants (*n* = 176)

Characteristic	*n* (%) or mean ± SD	PCS^a^	*p* ^b^	MCS^a^	*p* ^b^
**Total**	176 (100)	28.9 ± 17.1		47.9 ± 11.2	
***Sociodemographic characteristics***					
** Sex**			**0.002**		**0.023**
Male	78 (44.3)	24.4 ± 19.1		50.0 ± 11.6	
Female	98 (55.7)	32.5 ± 14.5		46.2 ± 10.7	
** Age (year)**	44.0 ± 14.6	*rho* = 0.270	**< 0.001**	*rho* = -0.200	**0.008**
** Marital status**			**0.004**		0.418
Single/divorced/separated/widowed	99 (56.2)	25.6 ± 18.8		48.5 ± 12.0	
Married/cohabit	77 (43.8)	33.1 ± 13.8		47.1 ± 10.1	
** Education**			0.682		0.535
Lower secondary or below	35 (19.9)	31.2 ± 16.0		45.7 ± 11.1	
Upper secondary	58 (33.0)	29.6 ± 15.6		49.0 ± 10.3	
Post-secondary	29 (16.5)	26.4 ± 17.1		47.1 ± 10.9	
Tertiary or above	54 (30.7)	28.0 ± 19.5		48.5 ± 12.4	
** Occupation**			**0.002**		0.425
Unemployed	57 (33.3)	22.9 ± 13.6		49.3 ± 10.9	
Full-time worker	54 (31.6)	33.6 ± 19.5		46.3 ± 12.6	
Part-time worker	22 (12.9)	26.5 ± 16.6		50.1 ± 8.1	
Retired	19 (11.1)	37.3 ± 12.4		44.4 ± 9.2	
Student	10 (5.9)	33.6 ± 19.0		48.5 ± 13.6	
Homemaker	9 (5.3)	33.8 ± 9.6		45.7 ± 12.1	
** Monthly household income (HK$)**			0.537		0.510
< 10,000	34 (19.8)	27.1 ± 19.1		47.3 ± 12.0	
10,000–29,999	75 (43.6)	27.8 ± 15.9		48.9 ± 10.1	
30,000–49,999	31 (18.0)	30.0 ± 18.9		45.4 ± 12.1	
> 50,000	32 (18.6)	32.5 ± 16.9		48.6 ± 12.7	
** Type of housing**			**< 0.001**		**0.011**
Public housing	85 (48.3)	22.5 ± 15.5		50.3 ± 10.4	
Private housing	59 (33.5)	36.5 ± 15.3		44.7 ± 10.0	
Others	32 (18.2)	32.0 ± 18.3		47.3 ± 13.9	
** Having domestic helper**			**0.024**		**0.004**
No	117 (66.5)	31.0 ± 17.5		46.2 ± 11.2	
Yes	59 (33.5)	24.8 ± 15.9		51.3 ± 10.6	
** Not receiving adequate treatment due to financial problem**			**0.001**		0.560
No	104 (59.1)	32.7 ± 16.4		47.2 ± 11.6	
Yes	51 (29.0)	22.6 ± 14.8		49.3 ± 10.5	
Unsure	21 (11.9)	25.1 ± 21.2		47.9 ± 11.4	
***Health-related conditions***					
** Activities of daily living**			**< 0.001**		**< 0.001**
Independence	77 (43.8)	39.5 ± 14.3		44.3 ± 9.8	
Slight dependence	11 (6.3)	32.3 ± 12.5		41.2 ± 13.3	
Moderate dependence	50 (28.4)	21.9 ± 14.6		49.6 ± 9.8	
Severe dependence	32 (18.2)	16.1 ± 13.4		54.1 ± 11.9	
Total dependence	6 (3.4)	14.1 ± 11.9		58.5 ± 9.7	
** Total number of disease (including rare and chronic disease)**	2.5 ± 1.5	*rho* = -0.056	0.458	*rho* = 0.148	**0.050**
** Sleep quality**			0.114		**0.023**
Good sleeper (PSQI ≤ 5)	79 (44.9)	31.2 ± 18.1		50.0 ± 10.9	
Poor sleeper (PSQI > 5)	97 (55.1)	27.1 ± 16.2		46.2 ± 11.3	
** Disability in community participation** (WHODAS 2.0 domain score)^c^	44.5 ± 22.5	*r* = -0.473	**< 0.001**	*r* = -0.010	0.899
***Patient experience***					
** Age at diagnosis (year)**	27.5 ± 17.7	*rho* = 0.192	**0.012**	*rho* = -0.170	**0.027**
** Time to diagnosis (year)**	5.6 ± 9.8	*rho* = -0.111	0.145	*rho* = 0.089	0.242
** Number of specialists visited to confirm diagnosis**			0.142		0.943
1	72 (40.9)	29.2 ± 16.5		47.6 ± 11.3	
2	38 (21.6)	30.6 ± 15.4		48.5 ± 10.0	
≥ 3	37 (21.0)	23.6 ± 18.2		48.4 ± 12.5	
Unsure	29 (16.5)	32.7 ± 18.9		47.0 ± 11.3	
** Number of misdiagnosis**			0.434		0.918
0	100 (56.8)	30.2 ± 17.7		48.4 ± 12.0	
1	27 (15.3)	29.1 ± 17.4		47.5 ± 10.4	
≥ 2	16 (9.1)	29.6 ± 15.6		47.9 ± 12.1	
Unsure	33 (18.8)	24.5 ± 15.9		46.8 ± 9.1	
** Experienced uncertainties and despair over the diagnosis**			0.135		**0.007**
Disagree	17 (9.7)	30.4 ± 17.6		50.8 ± 8.5	
Neutral	43 (24.4)	24.4 ± 16.9		51.9 ± 11.0	
Agree	116 (65.9)	30.4 ± 17.0		46.0 ± 11.2	
** Receiving enough information on managing condition**			0.526		0.080
Disagree	61 (34.7)	26.9 ± 17.7		46.7 ± 12.9	
Neutral	51 (29.0)	30.3 ± 16.9		46.2 ± 10.3	
Agree	64 (36.4)	29.6 ± 16.9		50.4 ± 9.9	
** Difficulty in receiving treatment for the rare disease**			0.254		0.416
Not difficult at all	51 (33.6)	31.3 ± 15.8		47.3 ± 10.4	
Neutral	35 (23.0)	26.0 ± 17.9		46.2 ± 9.2	
Difficult	66 (43.4)	26.6 ± 18.2		49.1 ± 12.2	
** Difficulty in receiving treatment for managing symptoms**			0.607		0.485
Not difficult at all	70 (47.6)	27.7 ± 16.4		48.3 ± 11.0	
Neutral	40 (27.8)	26.0 ± 19.3		48.7 ± 11.3	
Difficult	34 (23.6)	30.1 ± 17.9		45.7 ± 12.9	
** Satisfaction with treatment**			0.656		**0.016**
Dissatisfied	48 (27.3)	27.5 ± 18.1		44.6 ± 13.1	
Neutral	74 (42.1)	30.3 ± 16.0		47.8 ± 9.7	
Satisfied	54 (30.7)	28.3 ± 17.9		50.9 ± 10.7	
***Psychosocial support***					
** Social support** (MSPSS score)^d^	41.1 ± 10.4	*r* = 0.111	0.142	*r* = 0.113	0.136
** Being treated friendly over half of the time**			0.112		0.276
No	8 (4.6)	19.5 ± 24.3		43.7 ± 20.3	
Yes	168 (95.5)	29.4 ± 16.7		48.1 ± 10.7	
** Being understood over half of the time**			0.401		0.106
No	55 (31.3)	27.3 ± 15.7		45.9 ± 11.5	
Yes	121 (68.8)	29.6 ± 17.8		48.8 ± 11.0	
** Being respected over half of the time**			0.237		0.140
No	18 (10.2)	24.4 ± 17.1		44.2 ± 13.6	
Yes	158 (89.8)	29.4 ± 17.1		48.3 ± 10.9	
** Relationships with my family and friends suffered due to the disease**			**0.028**		0.282
Disagree	62 (35.2)	33.2 ± 16.9		49.0 ± 10.7	
Neutral	32 (18.2)	29.1 ± 17.4		49.4 ± 10.2	
Agree	82 (46.6)	25.6 ± 16.7		46.4 ± 11.9	
***Illness perception***					
** Feeling worried about future health**			0.391		**0.013**
Disagree	20 (11.4)	28.8 ± 18.2		54.6 ± 9.8	
Neutral	26 (14.8)	33.1 ± 12.9		45.6 ± 7.5	
Agree	130 (73.9)	28.1 ± 17.7		47.3 ± 11.7	
** Feeling hopeful that the disease will be cured**			0.627		0.531
Disagree	122 (69.3)	28.7 ± 17.0		47.3 ± 11.5	
Neutral	32 (18.2)	27.5 ± 17.8		48.8 ± 11.1	
Agree	22 (12.5)	32.0 ± 17.4		49.9 ± 10.1	
** Have accepted the illness and have a positive outlook on life**			0.601		**< 0.001**
Disagree	22 (12.5)	32.1 ± 18.9		37.3 ± 10.8	
Neutral	51 (28.9)	27.7 ± 16.3		45.6 ± 9.8	
Agree	103 (58.5)	28.8 ± 17.3		51.3 ± 10.3	
***Self-esteem***			0.405		**< 0.001**
Low self-esteem	39 (22.2)	26.9 ± 17.7		41.2 ± 12.1	
Within normal range	127 (72.2)	29.0 ± 16.8		49.4 ± 10.1	
High self-esteem	10 (5.7)	35.1 ± 19.9		54.3 ± 11.9	

Abbreviations: MCS, Mental Component Score; MSPSS, Multidimensional Scale of Perceived Social Support; PCS, Physical Component Score; PSQI, Pittsburgh Sleep Quality Index; SD, standard deviation; WHODAS, World Health Organization Disability Assessment Schedule

^a^ Results are mean ± SD or correlations with *r*-value (Pearson) or *rho*-value (Spearman)

^b^ *p*-values were from t-tests or ANOVA for categorical variables, and Pearson or Spearman correlation tests for continuous variables

^c^ WHODAS 2.0 score ranges from 0 to 100, where 0 = no disability, 100 = full disability

^d^ MSPSS score ranges from 12 to 60, where a higher score indicates higher level of social support

### Sample characteristics

Table 2 summarizes the descriptive statistics and univariate analyses of different variables with Physical Component Summary (PCS) and Mental Component Summary (MCS) scores. Overall, the mean PCS and MCS in this sample were 28.9 (SD 17.1) and 47.9 (SD 11.2) respectively. As both summary scores are norm-based with a mean of 50 in the general Hong Kong population where higher scores indicate better health [31, 32], the PCS and MCS scores suggest markedly poor physical health and compromised mental health. The Cronbach’s alpha was 0.698 for PCS and 0.758 for MCS.

#### Sociodemographic and socioeconomic characteristics

Age of the respondents ranged from 18 to 78, with a mean of 44.0 (SD 14.6). 55.7% of the respondents were female, 43.8% were married or cohabit, and 80.2% had attained at least upper secondary-level education. 44.5% were full-time or part-time workers, while 33.3% were unemployed. The majority of the respondents had a monthly household income between HK$10,000 and 29,999 (43.6%) and lived in public housing (48.3%). 66.5% did not have a domestic helper, and 29.0% responded that they did not receive adequate treatment due to financial problem. In the univariate analyses, it was observed that respondents who were male, younger, unmarried, unemployed, living in public housing, having domestic helper, and not receiving adequate treatment due to financial problem had lower PCS scores (*p* < 0.05); while male, older age, living in public housing, and having domestic helper were associated with higher MCS scores (*p* < 0.05).

#### Health-related conditions

In terms of activities of daily living (ADL), as assessed by the Barthel Index (scores range from 0 to 100, with higher scores indicating greater independence) [33], nearly half (43.8%) were classified as independent (score 100), while more than one-fifth (21.6%) were severely or totally dependent (score ≤ 60). The Cronbach’s alpha was 0.898 for the Barthel Index for ADL. PCS scores were lower with increased levels of dependence, but MCS scores were higher in more dependent individuals. This contrasting finding may reflect response shift or adaptation, whereby individuals with greater physical limitations may recalibrate their psychological health expectations. The mean total number of diseases a patient had at the same time is 2.5 (SD 1.5), and it showed a weak, albeit significant, positive correlation with MCS scores (*rho* = 0.148; *p* = 0.050).

Using the Pittsburgh Sleep Quality Index (PSQI), where scores above 5 indicate poor sleep quality [34], more than half (55.1%) of our respondents were classified as poor sleepers. These individuals had a lower mean MCS score compared with the good sleepers (*p* = 0.023). The mean PSQI score in this sample was 7.1 (SD 4.3), which exceeds the clinical cutoff of 5 and suggests substantial sleep disturbances. The Cronbach’s alpha was 0.762 for the PSQI. We also observed that individuals with higher disability in community participation, as indicated by higher World Health Organization Disability Assessment Schedule (WHODAS) 2.0 domain score (scores range from 0 to 100, with higher scores indicating greater disability) [35], tended to have lower PCS scores (*r*=-0.473; *p* < 0.001). The Cronbach’s alpha was 0.894 for the WHODAS 2.0.

#### Patient experience

On average, the respondents were diagnosed with their corresponding RDs at age 27.5 (SD 17.7), and it took them 5.6 years (SD 9.8) to confirm the diagnosis. Nearly half (42.6%) visited more than one specialist to receive a confirmed diagnosis, and nearly a quarter (24.4%) encountered at least one misdiagnosis. Over half (65.9%) experienced uncertainties and despair on their path to diagnosis, while more than one-third (34.7%) disagreed that they had received enough information on managing the condition. Of the sample, 43.4% and 23.6% of the sample found difficulties in receiving treatment for the RD and for managing symptoms respectively, and more than a quarter (27.3%) reported dissatisfaction with the treatment being received. Those with an older age at diagnosis had a higher PCS score (*rho* = 0.192; *p* = 0.012) but lower MCS score (*rho*=-0.170; *p* = 0.027). Experiencing uncertainties and despair over the diagnosis (*p* = 0.007), not receiving enough information on managing condition (*p* = 0.080), and dissatisfaction with treatment receiving (*p* = 0.016) were also associated with lower MCS scores in the univariate analyses. Also specifically, 83% thought that their daily lives have been negatively affected by their RDs, and 60.4% thought that their conditions had major or severe impact on their work. Close to half (48.4%) had to even quit their jobs due to their conditions.

#### Psychosocial support

The mean Multidimensional Scale of Perceived Social Support (MSPSS) score was 41.1 (SD 10.4) in our sample. As MSPSS scores range from 12 to 84, with scores of 12–35 categorized as low support, 36–60 categorized as medium support, and 61–84 as high support, our score indicates a medium level of perceived social support [36]. This variable showed no significant association with either PCS or MCS score. Regarding social interactions, 4.5%, 31.3% and 10.2% had the feeling of *not* being treated friendly, understood, and treated with respect by other people over half of the time, respectively. While only 35.2% disagreed that their relationships with family and friends suffered due to the disease, nearly half (46.6%) agreed that such relationships had suffered. These individuals had a significantly lower mean PCS score (*p* = 0.028). The Cronbach’s alpha was 0.898 for the Barthel Index for ADL.

#### Illness perception

Compared with those who were not worried about health in the future (11.4%), those who were worried (73.9%) had significantly lower MCS scores (*p* = 0.013). As high as 69.3% did not feel hopeful that their diseases would be cured in the future. Meanwhile, more than half (58.5%) agreed that they had accepted the illness and had a positive outlook on life, and these individuals had a higher mean MCS score than those who disagreed (*p* < 0.001).

#### Self-esteem

Using the Rosenberg Self-esteem Scale (RSE) (scores below 15 indicate low self-esteem, scores between 15 and 25 indicate normal range, and scores above 25 indicate high self-esteem) [37, 38], nearly a quarter (22.2%) of respondents had low self-esteem,72.2% were within normal range, and 5.7% had high self-esteem. Those with higher self-esteem had higher MCS scores (*p* < 0.001) in univariate analysis. The Cronbach’s alpha was 0.874 for the RSE.

### Multivariable linear regression analyses

Based on the results of univariate analyses, sociodemographic characteristics, health-related condition, patient experience, psychosocial support, illness perception, and self-esteem variables with *p* < 0.1 were included in linear regression models to assess their associations with PCS and MCS scores independently of other factors (Table 3). The PCS model included 11 variables, with an adjusted R-squared of 48%; while the MCS model included 14 variables, with an adjusted R-squared of 39%. It was observed that female (β = 8.01; *p* < 0.001) and older age (β per 1 year increase = 0.25; *p* = 0.023) were associated with higher PCS scores; while higher dependence in ADL (β for total dependence vs. independence=-21.66; *p* = 0.001) and higher disability in community participation (β=-0.15; *p* = 0.013) were associated with lower PCS scores. On the contrary, older age was associated with decreased MCS scores (β per 1 year increase=-0.19; *p* = 0.003), and a higher level of dependence was associated with increased MCS scores (β for total dependence vs. independence = 12.31; *p* = 0.004). In addition, acceptance of illness and having a positive outlook on life (β = 7.97; *p* = 0.002), and high self-esteem (β = 8.71; *p* = 0.018) also had significant positive associations with MCS scores.

**Table 3:** Multivariable associations of sociodemographic characteristics, health-related condition, patient experience, psychosocial support, illness perception and self-esteem with PCS and MCS scores

Characteristic	PCS (Adjusted *R*^2^ = 0.48)		MCS (Adjusted *R*^2^ = 0.39)	
	β (95% CI)^a^	*p*	β (95% CI)^a^	*p*
***Sociodemographic characteristics***				
** Sex**				
Male	0	-	0	-
Female	**8.01 (4.07**,** 11.95)**	**< 0.001**	-0.83 (-3.62, 1.96)	0.557
** Age (year)**	**0.25 (0.03**,** 0.47)**	**0.023**	**-0.19 (-0.32**,** -0.06)**	**0.003**
** Marital status**				
Single/divorced/separated/widowed	0	-	-	-
Married/cohabit	0.78 (-3.71, 5.26)	0.732	-	-
** Occupation**				
Unemployed	0	-	-	-
Full-time worker	-1.41 (-7.19, 4.36)	0.629	-	-
Part-time worker	-2.71 (-9.82, 4.39)	0.451	-	-
Retired	6.95 (-0.19, 14.09)	0.056	-	-
Student	2.56 (-7.94, 13.07)	0.630	-	-
Homemaker	-4.53 (-13.78, 4.71)	0.334	-	-
** Type of housing**				
Public housing	0	-	0	-
Private housing	4.46 (-0.19, 9.12)	0.060	-2.24 (-5.53, 1.06)	0.182
Others	3.89 (-1.73, 9.51)	0.173	-3.23 (-7.28, 0.82)	0.117
** Having domestic helper**				
No	0	-	0	-
Yes	1.99 (-2.76, 6.75)	0.409	1.33 (-1.98, 4.64)	0.428
** Not receiving adequate treatment due to financial problem**				
No	0	-	-	-
Yes	-4.41 (-9.09, 0.26)	0.064	-	-
Unsure	-0.21 (-6.50, 6.07)	0.947	-	-
***Health-related conditions***				
** Activities of daily living**				
Independence	0	-	0	-
Slight dependence	-3.83 (-11.83, 4.18)	0.346	0.66 (-5.27, 6.59)	0.826
Moderate dependence	**-12.59 (-17.89**,** -7.28)**	**< 0.001**	**6.01 (2.52**,** 9.50)**	**0.001**
Severe dependence	**-18.18 (-24.73**,** -11.63)**	**< 0.001**	**8.39 (3.83**,** 12.95)**	**< 0.001**
Total dependence	**-21.66 (-33.95**,** -9.38)**	**0.001**	**12.31 (4.08**,** 20.54)**	**0.004**
** Total number of diseases**	-	-	-0.23 (-1.22, 0.76)	0.643
** Sleep quality**				
Good sleeper (PSQI ≤ 5)	-	-	0	-
Poor sleeper (PSQI > 5)	-	-	-2.09 (-5.04, 0.85)	0.163
** Disability in community participation** (WHODAS score)	**-0.15 (-0.26**,** -0.03)**	**0.013**	-	-
***Patient experience***				
** Age at diagnosis (year)**	-0.04 (-0.19, 0.12)	0.630	0.03 (-0.07, 0.14)	0.532
** Experienced uncertainties and despair over the diagnosis**				
Disagree	-	-	0	-
Neutral	-	-	-1.53 (-6.99, 3.92)	0.580
Agree	-	-	-3.90 (-8.92, 1.13)	0.127
** Receiving enough information on managing condition**				
Disagree	-	-	0	-
Neutral	-	-	-1.84 (-5.39, 1.71)	0.308
Agree	-	-	-1.14 (-4.61, 2.34)	0.518
** Satisfaction with treatment**				
Dissatisfied	-	-	0	-
Neutral	-	-	2.04 (-1.49, 5.57)	0.255
Satisfied	-	-	3.29 (-0.39, 6.97)	0.079
***Psychosocial support***				
** Relationships with my family and friends suffered due to the disease**				
Disagree	0	-	-	-
Neutral	0.35 (-5.60, 6.30)	0.907	-	-
Agree	-1.00 (-5.80, 3.81)	0.682	-	-
***Illness perception***				
** Feeling worried about future health**				
Disagree	-	-	0	-
Neutral	-	-	-4.40 (-10.25, 1.44)	0.139
Agree	-	-	-2.16 (-7.03, 2.71)	0.382
** Have accepted the illness and have a positive outlook on life**				
Disagree	-	-	0	-
Neutral	-	-	4.20 (-0.80, 9.19)	0.099
Agree	-	-	**7.97 (2.90**,** 13.03)**	**0.002**
***Self-esteem***				
Low self-esteem	-	-	0	-
Within normal range	-	-	**5.51 (1.43**,** 9.58)**	**0.008**
High self-esteem	-	-	**8.71 (1.54**,** 15.90)**	**0.018**

Abbreviations: CI, confidence interval; PCS, Physical Component Score; MCS, Mental Component Score; PSQI, Pittsburgh Sleep Quality Index; WHODAS, World Health Organization Disability Assessment Schedule

^a^ All the listed variables are mutually adjusted in corresponding models

## Discussion

### Summary of key findings

To our knowledge, this is the first study in Hong Kong that quantifies the impact of RDs on patients’ health-related quality of life (HRQL), and comprehensively examines their experience throughout the disease journey. Our findings showed that RD patients in our sample generally experienced poor HRQL. They also faced multiple challenges and difficulties throughout their disease journey, notably delayed diagnoses, a lack of information on managing their RD conditions, and huge financial burdens.

### Poor physical and psychological health among RD patients

Our findings suggested that RD patients generally have poor physical health, which tends to be worse than that of the general population of Hong Kong. This result is consistent with studies in the US and the UK [11–13] which have similarly reported poorer physical health among RD patients, as well as with more recent international research demonstrating persistent physical health disparities among RD populations globally [41]. The mean PCS score in this study sample is 28.9 (SD 17.1), which is significantly lower than that observed for the general adult population in a previous Hong Kong household survey (mean 51.5; SD 9.0) [42], and even lower than that for Stage IV colorectal cancer patients in Hong Kong (mean 40.4; SD 12.5) [43]. Moreover, the mean PSQI score, which measures one’s sleeping quality, of our respondents is 7.1 (SD 4.3), which is higher than that of 5.3 (SD 3.3) in a population-based study in Hong Kong [44], implying a worse sleeping quality among RD patients compared with the general population in the city. RD patients of our sample also had higher physical disability level than some vulnerable groups in Hong Kong. For instance, the proportion of respondents who had a total ADL score equal to or below 60 (i.e., severely dependent or totally dependent in performing self-care activities) was 21.6%, which was higher than that of 19.0% in a previous study among older people living in nursing homes in Hong Kong [45]. In terms of participation of community activities, in comparison with a previous study in Hong Kong that adapted the WHODAS 2.0 questionnaire for patients with various types of disabilities and chronic illnesses, their mean score (mean 42.6; SD 26.1) was found to be lower than that of our rare disease patients sample (mean 44.5; SD 22.5) [46], indicating even greater disability among rare disease patients than other disabled and chronically ill non-RD patients.

Apart from poor physical health status, RD patients in our sample also had poor psychological health. Specifically, 22.2% of our respondents were found to have low self-esteem, which previous research has associated with a higher risk of other psychological health issues such as depression and anxiety [47]. The mean MCS (mean 47.9; SD 11.2) in this study sample was also found to be lower than that of the general population in another cross-sectional study in Hong Kong (mean 54.9; SD 8.5) [41], and even lower than that of Stage IV colorectal cancer patients (mean 54.3; SD 10.2) [42]. This evidence suggests that RD patients may be more likely to have worse psychological well-being as compared with the general population. Nevertheless, our findings also showed that a more positive illness perception and higher self-esteem of RD patients tended to be two factors that positively affected their psychological health status, which was in line with previous studies suggesting that patients with greater acceptance and optimism tend to have improved psychological well-being, thus improving one’s HRQL [9, 48].

### Challenges along the disease journey: diagnosis, information, and financial burden

Our study findings also depicted challenges and difficulties that RD patients faced throughout their disease journey. First, RD patients in our sample had been subject to delays in diagnosis, in which the average time of 5.6 years to receive a proper diagnosis was highly similar to that in the UK but shorter than that in the US (7.6 years) [19]. Moreover, 34.7% of our respondents reported that they had not received sufficient information on managing their conditions. This finding was in line with previous research findings suggesting that RD patients tend to receive insufficient information on their RD(s) during their disease journey [10, 11, 20].

Another difficulty that RD patients could face throughout their disease journey as depicted in our study was related to their financial burdens. 29.0% of our RD respondents thought that they did not receive adequate treatment due the lack of financial resources, which was significantly greater than the 8.4% observed in the general population of Hong Kong [46]. Our study also shows that those who did not receive adequate treatment due to lack of financial means also had lower PCS scores. This result was consistent with other existing findings in the literature, suggesting that being unable to seek healthcare due to a lack of financial means tends to be associated with poorer health [49–52]. Our evidence implied that the goal of the HKSAR government’s long-established overarching healthcare policy that *“no one should be denied adequate healthcare through lack of means”* [53] was not met, despite the non-denial commitment and heavy financial subsidy provided by the government in the public healthcare sector. This is a significant finding because the overarching policy was established based on the concept of universal health coverage; nevertheless, empirical evidence shows that despite universal coverage, there are people who were denied, albeit inadvertently, the healthcare that they need due to lack of financial means [49]. This echoes the position paper, published by Rare Disease International, in which they advocated for the inclusion of rare diseases in the global framework of universal health coverage [54]. Moreover, our study findings suggest that the proportion of RD patients who are denied adequate healthcare may be significantly higher than that of the general population, implying the presence of severe health inequalities in Hong Kong. If indeed no one were denied adequate healthcare due to lack of means, it would have been an ideal and highly welcomed situation; however, that is not the reality. In other words, the issue lies not with the policy itself, but with its implementation.

### Policy implications: moving beyond universal health coverage

To address the health inequalities RD patients face, first, further enhancement and refinement of existing healthcare policy instruments should be made. For instance, since we knew from our findings that the livelihood and economic activities of many RD patients had been greatly affected, thereby creating financial burdens for them, the government should try to alleviate these burdens. As mentioned in our background above, Samaritan Fund and Community Care Fund were established to help financially support patients who need drug and non-drug items that are expensive and thus not covered as standard items in the public healthcare sector. Nevertheless, patients in the past still complained that their finances were not adequately protected in these two funding schemes because they were still required to contribute significantly out of their own pockets [55]. In response, amendments in the means-test mechanism were made to lower RD patients’ out-of-pocket contribution by only accounting for 50% (instead of the original 100%) of net assets of the patients’ households towards estimating their annual disposal financial resources, and to further remove non-monetary barriers to access services and relieve financial and emotional burden from these patients and their families by including only the assets of the patients and their core family members living under the same roof in the calculation of contribution [56]. Preliminary findings showed that both the number of applications and subsidy amount granted to non-social security recipients had increased, as had the percentage of approved applications for households of one to two people. Nevertheless, a post-implementation review should be conducted to evaluate whether further amendments can be made, especially since the current schemes still do not address the extra burden of multiple applications from patients within the same households given that many RDs tend to be genetically linked. On the other hand, under the current dual-track public-private healthcare system in Hong Kong, the public sector, which is heavily subsidized by the government, provides approximately 90% of inpatient services [57]. Many patients would choose to receive medical services from the public sector due to its lower medical cost [58], which, however, drives up the demand of such services and leads to a long waiting time [59]. To shorten the waiting time for diagnosis of RD patients, policymakers may consider strengthening the Public-Private Partnership Program where RD patients could receive diagnoses in the private healthcare sector, while the fees are subsidized by the government.

However, to get to the bottom of the issue of health inequalities, one must also look beyond the usual healthcare policies, as emphasized by the social determinants of health (SDH) framework [60]. This is because many of the SDH that lead to health inequalities exist outside of the healthcare settings, and they may be barriers to accessing adequate healthcare for the RD patients. As shown in this study, many other facets of life (including but not limited to economic activities, ability to work, livelihood, and other psychosocial aspects) were affected by the RD conditions. Policies that influence the social aspects of RD patients and their family members must also be considered if society truly espouses to reduce health inequalities faced by these individuals. While the existence of a public healthcare sector in Hong Kong ensures universal health coverage, our findings showed that this does not necessarily translate into true equality of opportunity for accessing services when needed. This is largely due to the overarching healthcare policy in Hong Kong, which adopts a resourcist approach that emphasizes individual rights and entitlements but stops short of ensuring equitable access of social goods. In other words, this type of policy does not adequately account for differences in health, needs, and capabilities in converting these resources into the valuable outcomes for these patients [61, 62].

### A call for a capability-based approach

This brings us to a final point we wish to emphasize, which has international relevance beyond Hong Kong. The key message of this study is that simply adopting a resource-focused healthcare policy for rare disease patients is insufficient. The typical healthcare system often remains passive, relying on patients to seek treatment rather than proactively reaching out to the community. To effectively address the challenges faced by rare disease patients, governments must therefore go beyond merely ensuring universal health coverage; this is especially crucial in contexts where healthcare policies aimed at benefiting rare disease patients are currently under consideration. Rare disease patients experience unique struggles, such as delayed diagnoses, frequent misdiagnoses, constant uncertainties in treatment, and a profound impact on nearly every aspect of their lives. They also often encounter lower societal awareness, understanding and support for their diseases, as well as a heightened dependence that places a great burden on their family members. Given these difficulties, an overarching one-size-fits-all healthcare policy that emphasizes universal coverage alone would fall short. Instead, a comprehensive rare disease policy that extends beyond mere distributive justice to encompass broader social justice is critical. One such approach is the capabilities theory as advanced by Nussbaum and Sen [63], which focuses on individuals’ actual capability to achieve well-being rather than solely their rights or entitlements.

In light of this framework, we propose the establishment of a comprehensive rare disease policy in Hong Kong that not only addresses essential health care needs – such as diagnosis, treatment, medication, and rehabilitation – but also tackles the broader social determinants of health that exist outside healthcare settings. These determinants include but are not limited to social welfare, financial support, community engagement, education, employment opportunities, as well as efforts to transform the sociocultural environment to promote awareness, understanding, and compassion for the rare disease communities. This comprehensive approach is increasingly recognized in the global rare disease policy literature as essential for addressing the multifaceted needs of this population [64, 65]. Achieving this vision will require collaboration that extends beyond traditional health departments to include various non-health government agencies, non-governmental organizations, patient groups, and caregivers.

### Limitations

There are limitations to this study. First, sampling this population is challenging due to the absence of a RD patient registry in Hong Kong. Many RD patients who have severe disabilities were not included in the sample population as they were physically confined to their own residence or communities. To overcome this difficulty, the present study employed a mixed-mode survey approach, utilizing the network of a patient organization that also includes a regular membership of RD patients. Consequently, our sample was not randomly selected, prompting caution in interpreting the estimated proportions as truly representative of the prevalence within the broader population. As such, the potential sampling bias could go in both directions. On one hand, participants who were able to show up to the study setting and complete the survey tended to be healthier, more active, and less impaired, thereby leading to an underestimation of poor health outcomes in our analysis. On the other hand, we could have also under-sampled rare disease patients with relatively mild impairments, as they might feel less compelled to express their concerns and frustrations regarding the system and their situations through our study. As a result, we believe that our study findings primarily reflect the experiences of patients situated toward the middle of the distribution of the actual rare disease patient population. In relation to this, although our sample may be relatively small and the data were collected some years ago, our study still makes a significant contribution by offering a detailed examination of the health-related quality of life and other social challenges among individuals with rare diseases in Hong Kong. While we recognize the potential benefits of using a more current sample for our research, we believe that significant changes in the sociocultural and healthcare landscape for rare disease patients in Hong Kong have not happened, particularly in the absence of an established rare disease policy. Future studies will be necessary to provide deeper insights into the evolving situation once significant changes in rare disease policy are implemented in Hong Kong.

Second, there were possibility of recall bias since the questions were self-reported, and the study design was cross-sectional in nature. Third, development of more specific questions for RD population in the future is required for our study as the pre-existing instruments used in the survey were not specifically developed for them, whose needs and experiences may be different and unique from the general population. Fourth, the nature of various RDs is diverse. Therefore, one should be careful in over-generalizing the findings of this study to specific RDs. Although, the etiology and symptoms may vary in different RDs, RD patients in general often share similar experiences of delayed diagnosis, misdiagnosis, and lack of effective treatments [17]. Comparative analysis by each rare disease is a direction that can be taken in the future, especially if an official comprehensive patient registry was to be established. Fifth, our study focused on individuals over 18 years or above, thereby excluding those whose rare disease conditions have already manifested during childhood or adolescence. While the survey was not designed for children, future studies can target family members, such as parents, of children affected by rare diseases for a more comprehensive understanding of the broader impact of rare disorders. Sixth, our current study focused on rare disease patients and did not compare this group with individuals affected by non-genetic psychological disorders or non-rare chronic disorders, such as oncological conditions. Future research could address this gap to provide additional insights into the physical and psychological health outcomes of rare disease patients in comparison to other patient groups. Last, since the questions were quantitatively measured, no in-depth qualitative information concerning the different needs and experiences of RD patients were collected. Further investigation could be conducted using qualitative methods in the future.

## Conclusion

This study confirms that RDs generally result in poor physical and psychosocial health and pose challenges in RD patients’ daily lives beyond impact on their health, including healthcare experience, social life, psychosocial support, illness perception, self-esteem, and financial burdens. Due to the multi-faceted impact of RD on their patients, the authors opine that it is critical to adopt a capability approach that does not merely emphasize on the resourcist right and entitlement of the patients, but also ensures equality in capabilities in converting resources to outcomes they value by focusing on the social determinants of health beyond healthcare policies.

## Supplementary Information

Below is the link to the electronic supplementary material.

Supplementary Material 1

## Data Availability

The datasets generated and/or analyzed during the current study are not publicly available due to agreement with the subjects to safeguard confidentiality but are available from the corresponding author on reasonable request.
