# Supplementary material for: Health and social challenges faced by rare disease patients in Hong Kong: a cross-sectional study
Source: Orphanet J Rare Dis. 2026 Jul 24;21:371. doi: 10.1186/s13023-026-04482-4 (PMC13692896; doi:10.1186/s13023-026-04482-4)
Supplement: Supplementary file 1 [file 13023_2026_4482_MOESM1_ESM.docx]

**Appendix Table 1.** Sources of participant referrals for the study

Name of organization (*in alphabetical order*)

Child's Vision

Families of SMA Charitable Trust HK

FAP Patient Concern Group

Rare Disease Hong Kong (formerly Hong Kong Alliance for Rare Diseases)

Hong Kong Angelman Syndrome Foundation

Hong Kong Eye Hospital

Hong Kong Mucopolysaccharidoses & Rare Genetic Diseases Mutual Aid Group

Hong Kong Neurofibromatosis Association

Hong Kong Neuro-Muscular Disease Association

Hong Kong Red Cross Princess Alexandra School

Hong Kong Rett Syndrome Association

Hong Kong Spinocerebellar Ataxia Association

Little Life Warriors Society

Little People of Hong Kong

PNH Patient Right Concern Group

Primary Immunodeficiency League

Retina Hong Kong

Special Clinic (Paediatrics & Medicine) under Public Hospitals (Hospital Authority)

Systemic Vasculitis Mutual Support Group

The Hong Kong Association Of Myasthenia Gravis

The Philanthropic Community Pharmacy

Tuberous Sclerosis Complex Association of Hong Kong

Uveitis Fellows

Wilson's Disease Hong Kong

**Appendix Table 2.** Type of rare disease by sex (n=176)

| Type of rare disease^ | Overall | | Male | | Female | |
| --- | --- | --- | --- | --- | --- | --- |
|  | Number | % | Number | % | Number | % |
| Spinocerebellar ataxia | 29 | 16.5 | 16 | 20.5 | 13 | 13.3 |
| Multiple sclerosis | 14 | 8.0 | 4 | 5.1 | 10 | 10.2 |
| Neurofibromatosis | 14 | 8.0 | 4 | 5.1 | 10 | 10.2 |
| Retinitis pigmentosa | 14 | 8.0 | 8 | 10.3 | 6 | 6.1 |
| Spinal muscular atrophy | 14 | 8.0 | 6 | 7.7 | 8 | 8.2 |
| Muscular dystrophy | 13 | 7.4 | 8 | 10.3 | 5 | 5.1 |
| Myasthenia gravis | 12 | 6.8 | 3 | 3.8 | 9 | 9.2 |
| Skeletal dysplasia | 7 | 4.0 | 1 | 1.3 | 6 | 6.1 |
| Non-infectious non-anterior uveitis | 6 | 3.4 | 3 | 3.8 | 3 | 3.1 |
| Charcot-Marie-Tooth disease | 5 | 2.8 | 2 | 2.6 | 3 | 3.1 |
| Fabry disease | 5 | 2.8 | 2 | 2.6 | 3 | 3.1 |
| Transthyretin familial amyloid polyneuropathy | 5 | 2.8 | 0 | 0.0 | 5 | 5.1 |
| Achondroplasia | 4 | 2.3 | 1 | 1.3 | 3 | 3.1 |
| Takayasu arteritis | 4 | 2.3 | 0 | 0.0 | 4 | 4.1 |
| Mucopolysaccharidoses | 3 | 1.7 | 2 | 2.6 | 1 | 1.0 |
| Pompe disease | 3 | 1.7 | 3 | 3.8 | 0 | 0.0 |
| Aplastic anaemia | 2 | 1.1 | 1 | 1.3 | 1 | 1.0 |
| Behçet's disease | 2 | 1.1 | 1 | 1.3 | 1 | 1.0 |
| Glycogen storage disease | 2 | 1.1 | 1 | 1.3 | 1 | 1.0 |
| Osteogenesis Imperfecta | 2 | 1.1 | 0 | 0.0 | 2 | 2.0 |
| Paroxysmal nocturnal hemoglobinuria | 2 | 1.1 | 1 | 1.3 | 1 | 1.0 |
| Wilson's disease | 2 | 1.1 | 2 | 2.6 | 0 | 0.0 |
| X-linked thrombocytopenia | 2 | 1.1 | 2 | 2.6 | 0 | 0.0 |
| Angelman syndrome | 1 | 0.6 | 1 | 1.3 | 0 | 0.0 |
| Atrial septal defect | 1 | 0.6 | 0 | 0.0 | 1 | 1.0 |
| Churg–Strauss syndrome | 1 | 0.6 | 0 | 0.0 | 1 | 1.0 |
| Cone-rod dystrophy | 1 | 0.6 | 0 | 0.0 | 1 | 1.0 |
| Fibrodysplasia ossificans progressiva | 1 | 0.6 | 0 | 0.0 | 1 | 1.0 |
| Hunter syndrome/ MPS II | 1 | 0.6 | 1 | 1.3 | 0 | 0.0 |
| Idiopathic pulmonary fibrosis | 1 | 0.6 | 1 | 1.3 | 0 | 0.0 |
| Kennedy disease | 1 | 0.6 | 1 | 1.3 | 0 | 0.0 |
| Larsen syndrome | 1 | 0.6 | 0 | 0.0 | 1 | 1.0 |
| Leber's hereditary optic neuropathy | 1 | 0.6 | 1 | 1.3 | 0 | 0.0 |
| Motor neurone disease | 1 | 0.6 | 1 | 1.3 | 0 | 0.0 |
| Mucopolysaccharidosis IV | 1 | 0.6 | 0 | 0.0 | 1 | 1.0 |
| Neuromyelitis optica spectrum disorders | 1 | 0.6 | 0 | 0.0 | 1 | 1.0 |
| Peutz–Jeghers syndrome | 1 | 0.6 | 1 | 1.3 | 0 | 0.0 |
| Pierre Robin syndrome | 1 | 0.6 | 1 | 1.3 | 0 | 0.0 |
| Prader–Willi syndrome | 1 | 0.6 | 1 | 1.3 | 0 | 0.0 |
| Primary immunodeficiency | 1 | 0.6 | 1 | 1.3 | 0 | 0.0 |
| Schwannoma | 1 | 0.6 | 1 | 1.3 | 0 | 0.0 |
| Scleroderma | 1 | 0.6 | 0 | 0.0 | 1 | 1.0 |
| Stevens–Johnson syndrome | 1 | 0.6 | 0 | 0.0 | 1 | 1.0 |
| Undiagnosed disease | 1 | 0.6 | 0 | 0.0 | 1 | 1.0 |
| Usher syndrome | 1 | 0.6 | 1 | 1.3 | 0 | 0.0 |
| Wolfram syndrome | 1 | 0.6 | 0 | 0.0 | 1 | 1.0 |
| Overall | **189** | **100** | **83** | **100** | **106** | **100** |

^ This exceeds 176 since 163 (92.6%) had reported having one RD, while 13 (7.4%) had two RDs at the same time.
